# A systematic review and meta-analysis: probiotics in the treatment of irritable bowel syndrome

**DOI:** 10.1186/1471-230X-9-15

**Published:** 2009-02-16

**Authors:** Nourieh Hoveyda, Carl Heneghan, Kamal R Mahtani, Rafael Perera, Nia Roberts, Paul Glasziou

**Affiliations:** 1 Department of Primary Health Care, Centre for Evidence Based Medicine, University of Oxford, Rosemary Rue Building, Old Road Campus, Headington, Oxford, OX3 7LF, UK; 2 University of Oxford Health Care Libraries, Old Road Campus Library, Old Road Campus, Oxford, OX3 7LF, UK

## Abstract

**Background:**

Irritable Bowel Syndrome (IBS) is a common chronic gastrointestinal disorder and the evidence for efficacy of most drug therapies in the treatment of IBS is weak. A popular alternative is probiotics, which have been used in several conditions. including IBS. Probiotics are live microbial food supplements.

The aim of this systematic review and meta-analysis of randomized trials study was to evaluate the efficacy of probiotics in alleviating symptoms in patients with irritable bowel syndrome. We searched Ovid versions of MEDLINE (1950–2007), EMBASE (1980–2007), CINAHL (1982–2007), AMED (1985–2007), the Cochrane library and hand searched retrieved papers.

**Results:**

We identified 14 randomized placebo controlled trials. Combined data suggested a modest improvement in overall symptoms after several weeks of treatment: for dichotomous data from seven trials the overall Odds Ratio (OR) was 1.6 (95% CI, 1.2 to 2.2); for continuous data from six trials the standardised mean difference (SMD) was 0.23 (95% CI, 0.07 to 0.38).

For individual symptoms the results differed between the pooled dichotomous and pooled continuous data. Trials varied in relation to the length of treatment (4–26 weeks), dose, organisms and strengths of probiotics used.

**Conclusion:**

Probiotics may have a role in alleviating some of the symptoms of IBS, a condition for which currently evidence of efficacy of drug therapies is weak. However, as IBS is a condition that is chronic and usually intermittent longer term trials are recommended. Such research should focus on the type, optimal dose of probiotics and the subgroups of patients who are likely to benefit the most.

## Background

Irritable Bowel Syndrome (IBS) is a common chronic gastrointestinal disorder, characterized by abdominal pain, bowel dysfunction and bloating in the absence of structural abnormality [1]. In the West, about 15% of the population is affected at some time during their life, and it is more common in females [2]. IBS is also recognized in children [3]. The burden of illness associated with IBS is considerable. A UK study [4,5] found that IBS sufferers reported substantially lower quality of life scores (as measured by the SF36 health survey questionnaire). They had more time off work and used healthcare more often. IBS accounts for 12% of visits to primary care physicians and 28% of visits to gastroenterologists [6,7].

Patients with IBS may be classified by their predominant bowel habit: diarrhoea-predominant IBS, constipation-predominant IBS, or IBS with alternating bowel movements [8]. Diagnostic criteria such as Manning, Rome I, II and III have proved useful for research purposes by ensuring homogeneity of patient populations, though their applicability in clinical practice is limited and they are seldom used [1].

The evidence for efficacy of most drug therapies in the treatment of IBS is weak [9]. A popular alternative is probiotics, which have been used in several conditions [10,11] including IBS. Probiotics are live microbial food supplements. Examples include lactic acid bacteria and bifidobacteria which are widely used in yogurts and other dairy products. They retain viability during storage and survive passage through the stomach and small bowel [12]. The colonic microflora normally presents a barrier to invading organisms. However, when the integrity of the microbiota is impaired through illness, stress, antibiotics treatment, physiological alterations in the gut, or change in diet, pathogens may become established [12]. Bifidobactetrium resist the colonization of pathogens in the large bowel [13]. We aimed to assess whether probiotics alleviated symptoms in patients with IBS.

## Methods

### Literature search

We searched Ovid versions of MEDLINE (1950–2007), EMBASE (1980–2007), CINAHL (1982–2007), AMED (1985–20007), as well as the Cochrane Database of Systematic Reviews and Cochrane Controlled Trials Register, Cochrane Library issue 3, 2007 (search date August 2007). MESH terms used were 'PROBIOTICS' and 'COLONIC DISEASE', 'FUNCTIONAL' or 'IRRITABLE BOWEL SYNDROME'. Further terms were included as text words. A high sensitivity "therapy" (trials) filter was applied to the EMBASE search. No other limits were applied to any of the searches. All registers on Current Controlled Trials http://www.controlled-trials were searched to locate ongoing studies and, where possible, lead researchers were contacted for further details. In addition, we hand searched the reference lists of retrieved full-text papers.

### Selection

We included randomised controlled trials that compared the effects of any probiotic therapy (regardless of type, dose and duration of treatment) with placebo in patients with IBS. Studies were included only if the two groups were treated equally except for the provision of the probiotic to one group. Studies not adhering to this were excluded. For example, in Bittner's study [14] a two week randomised placebo control trial patients in the intervention group received a pro-biotic plus a pre-biotic. We included studies of adults and children with IBS consistent with Manning or Rome diagnostic criteria, which has recently been updated to Rome III [15].

The primary outcome measure was improvement in overall symptoms as defined by the presence or absence of the following physical symptoms: pain, flatulence, bloating, anxiety and quality of life. As a number of studies included symptoms of flatulence this was included in the analysis.

Secondary outcome measures were the following individual symptoms: pain, flatulence, bloating, anxiety and quality of life. Two reviewers independently assessed articles and abstracts and each put forward articles for inclusion. Non English language publications were excluded.

### Data abstraction

The review was carried out in accordance with the recommendations of the QUOROM statement [16]. Two reviewers independently assessed trial methodological quality and extracted data. Disagreements were resolved by discussion. All studies were assessed for methodological quality in four specific areas: method of randomisation, clear allocation concealment, blinding and use of intention to treat analysis. Total scores were given for each study included in the review, with maximum score of four. Data was also extracted on the number of participants, age range, diagnostic criteria used for IBS, study subgroups, the type and dose of probiotics, length of treatment and the relevant outcome measures used in the study. Study authors were contacted for missing or incomplete information.

### Data analysis

Review Manager Version 4.3.1 was used for the statistics analysis and odds ratio (OR) and 95% confidence interval (CIs) as summary statistics were calculated. A fixed-effects model with Mantzel-Haenzel method was used to calculate the pooled OR. For continuous outcomes reported on non-standard scales, standardized mean difference (SMD) were used. For studies with more than one intervention arm a conservative estimate (i.e. the intervention with the least effect) was used. Heterogeneity was examined with I^2 ^statistics and where important heterogeneity existed reasons for this were explored. Publication bias was examined by a funnel plot. A sensitivity analysis was carried out by excluding studies of lower quality (scores less than four) and subgroup analysis was undertaken for adults only and children only. Due to limited data, it was not possible to carry out a subgroup analysis for type of probiotics.

## Results

**Figure 1:**
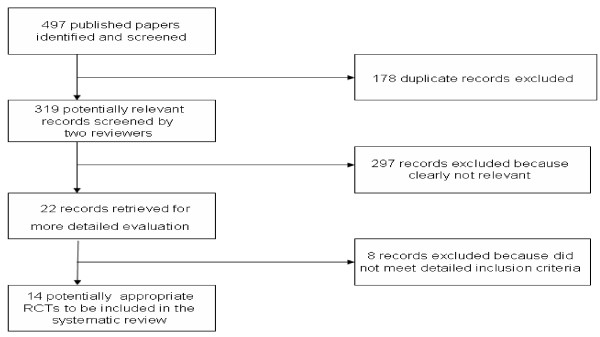
**Flow chart for search results**.

We identified 497 citations, 178 of which were duplicate records and were therefore excluded. Two authors screened 319 abstracts and identified potentially relevant articles. Twenty two retrieved articles were independently reviewed for inclusion and exclusion criteria. The reviewers were not masked to any aspect of the studies (e.g. journal type, author's names etc). On further analysis of retrieved articles, 8 trials were excluded for being non-controlled studies. Therefore, a total of 14 articles met the inclusion criteria [17-30] (figure. 1). As a number of studies included symptoms of flatulence this was included in the analysis. For example, in Gade's study [18] published in 1989, the IBS definition of constipation and/or diarrhoea, abdominal pain, meteorism, borborygmus and flatulence was used.

Trials were from USA (three), Poland (two), Ireland (two), UK (one) France (one), Israel (one), Finland (one), Italy (one), Sweden (one) and Denmark (one). One trial included women only [30]; the remainder included female and male participants with the majority being women (table 1). Two studies included children [17,19], though Bausserman's study [17] included an age range of 6–20 years. Trials varied in relation to the type, dose and strengths of probiotic(s) used. Number of probiotics varied from one to multiple, e.g. VSL#3 (contains 8 different bacterial strains). Three studies included more than one arm in the intervention group[27,29,30] (table 1).

**Table 1:** Study characteristics

First author	Number of participants (age range)l	Diagnostic criteria	Probiotics*	Measured outcomes included:	Length of treatment
Guyonnet, 2007, France	274 (20–65)	Rome II Constipation- Predominant IBS	Activia Danone, **Combination **containing Bifidobacterium. animalis DN 1730101(1.25 × 10^10 ^cfu per pot) together with S. thermophilus and L. bulgaricus 1.2 × 10^9 ^cfu per pot	Health related quality of life Bloating, abdominal pain, global digestive symptom	6 weeks
Gawronska A, 2007, Poland	37 (6–16)	Rome II	LGG (Lactobacillus rhamnosus GG) 3 × 10^9 ^cfu, twice daily,	Self reported abdominal pain	4 weeks
Whorwell PJ, 2006, UK	362 (19–69) All female	Rome II	3 different strengths treatments Bifidobacterium (B.) infantis 35624 × 10^6 ^live bacterial cells B. infantis 35624 × 10^8 ^live bacterial cells B. infantis 35624 × 10^10 ^live bacterial cells Once daily capsule	Global assessment (SGA) of IBS symptoms Abdominal pain/discomfort, Bloating/distension, passage of gas	4 weeks
Bausserman M, 2005, USA	64 (6–20)	Rome II	lactobacillus GG 10^10 ^and Inulin was also present both in treatment and in placebo one capsule twice a day	Changes in abdominal pain severity	6 weeks
Niv, 2005, Israel	54 (19–70)	Rome II	1 × 10^8 ^cfu of Lactobacillus reuteri ATCC 55730, four tablets to be taken for seven days, followed by a dose of 2 tablets per day until close of the study	abdominal pain, quality of life	6 months
Kajander K, 2005, Finland	103 (21–65)	Rome I and Rome II	**Combination** Lactobacillusrhamnosus GG, Lactobacillus rhamnosus LC 705, Bifidobacterium breve Bb 99 and Propionibacterium freudenreichii ssp. Shemanii JS 8–9 × 10^9^	Abdominal pain, distension, flatulence, borborygmi	6 months
Kim HJ, Vazquez Roque M, 2005, USA	48 (21–75)	Rome II	VSL # 3 mixture of bacteria **combination **twice daily (450 billion viable lyophilized bacteria) One packet twice a day	Abdominal bloating, flatulence, abdominal pain	8 weeks
O'Mahony L, 2005, Ireland	80 (18–73)l	Rome II	Either B infantis 35624 Or L. salivarIus UCC 4331 each delivered in a dose of 1 × 10 ^10^ Once Daily	Abdominal pain or discomfort, bloating or distension and bowel movement difficulty Quality of life assessment using an IBS specific questionnaire	8 weeks
Saggioro A, 2004, Italy	Unclear (6–64)	Rome II	**Combination **L. plantarum LP01 plus one strain of B. breve BR0 5 × 10 ^10 ^cfu/ml once a day **Combination **Strain of L. plantarum LP01 plus a strain of L. acidophilus LA02 one strain of B. breve BR0 5 × 10^9 ^cfu/ml once a day	Pain score at different locations in RLQ and LLQ of the abdomen Overall symptom score	4 weeks
Kim HJ, Camilleri M, 2003, USA	25 (19–70)	Rome II Diarrhea predominant symptom	**Combination **VSL # 3 mixture of bacteria One packet twice a day	abdominal pain, bloating, flatulence	8 weeks
Niedzielin K, 2001, Poland	40 (27–63)	Manning	Lactobacillus plantarum 299V 5 × 10^7 ^CFU/ml twice a day	improvement in pain and flatulence	4 weeks
Nobaek S, 2000, Sweden	60 (21–78)	Rome	400 ml/day of rose hip drink syrup containing 5 × 10 ^7 ^cfu/ml of L. plantarum DSM 9843 (strain 299V) and 0.009 g/ml oat flour	Overall GI function abdominal pain, flatulence, defecation	4 weeks
O'Sullivan MA, 2000, Ireland	24 (24–60)	Rome	Lactobacillus GG 1 × 10^10 ^cfu/day two tablets twice a day	Abdominal bloating, pain, bowel frequency	20 week
Gade, 1989, Denmark	54 (16–60)	constipation and or diarrheoa, abdominal pain, meteorism, borborygmus and flatulence	Paraghurt (freeze dried culture of Streptococcus faecium) 4 tablets morning and evening	abdominal pain, meteorism, borborygmus, flatulence	4 weeks

cfu: Colony Forming Unit

**Combination **denotes a mixture of probiotics

In six studies the length of treatment was four weeks, in three studies eight weeks, in two studies six weeks and in two studies six months (table 1). One study was a cross over of 20 weeks (two weeks wash in and washout) [28]. Diagnostic criteria for IBS used were as follows: ten trials used Rome II, two trials used Rome, and one trial used Manning. However, in Gade's study [18] published in 1989, the IBS definition of constipation and/or diarrhoea, abdominal pain, meteorism, borborygmus and flatulence was used. One study included constipation predominant IBS [20]; one included diarrhea-predominant symptom [22].

Measured outcomes varied, from overall gastrointestinal (GI) function to individual symptoms such as abdominal pain, flatulence and bloating. Four studies included quality of life using different questionnaires: Kajander et al [21] used RAND 36 item health survey [31] Guyonnet et al [20] used FDDQL questionnaire [32]. Whorwell [30] and O'Mahony [27] used IBS specific quality of life questionnaire [33].

### Assessment of quality of studies

Methodological qualities varied (Table 2), with seven studies scoring two or less and four studies scoring a maximum of four.

**Table 2:** Assessment of methodological quality of randomised trials:

Paper	Nobaek 2000	O Sullivan 2000	Niedzelin 2001	Kim 2003	Saggioro 2004	Kajander 2006	O' mahoney 2005	Gawronska 2007	Bausserman 2005	Whorwell 2006	Niv 2005	Kim 2005	Guyonnet 2007	Gade 1989
Randomization	-	-	-	-	-	+	+	+	+	-	-	-	+*	+
Concealment of Allocation	-	+	-	-	-	+	+	+	+	-	-	-	+*	+
Double Blinding	+	+	-	+	-	+	+	+	+	+	+	+	+*	+
Intention to treat	-	+	+	+	+	-	+	+	-	+	+	+	+	+
Total	1	3	1	2	1	3	4	4	3	2	2	2	4*	4

+ Clear reporting of the methodological quality

- Not clear from the paper

* Clarified after emailing author

### Overall improvement

**Figure 2:**
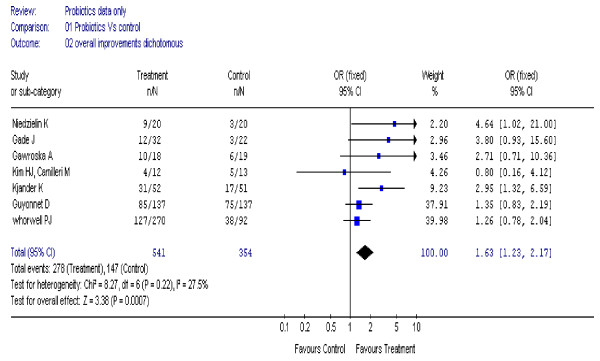
**Forest plot of improvement in overall symptoms (dichotomous data) in patients with IBS treated with probiotics compared to placebo**.

**Figure 3:**
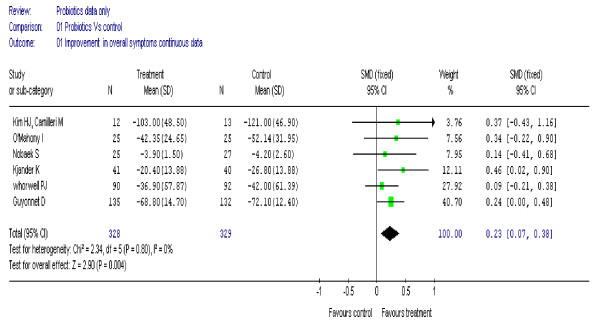
**Forest plot of improvement in overall symptoms (continuous data) in patients with IBS treated with probiotics compared to placebo**.

Combined data suggested a modest improvement in overall symptoms after several weeks of treatment: for dichotomous data from seven trials [18-22,24,30] (895 participants) (figure 2) the overall Odds Ratio (OR) was 1.6 (95% CI, 1.2 to 2.2); heterogeneity I^2 ^= 28%. For continuous data from six trials (657 participants) [20-22,26,27,30], the standardised mean difference (SMD) was 0.23 (95% CI, 0.07 to 0.38), I^2 ^= 0% (figure 3).

In the six trials [18,20-22,24,30] of adults only (850 participants) the overall improvement in symptoms remained statistically significant, OR 1.59 (95% CI, 1.19 to 2.13), I^2 ^= 35%. Results for overall improvement in symptoms were stable to sensitivity analysis [18-20]; (365 participants), OR 1.62 (95% CI, 1.06 to 2.48), I^2 ^= 20%.

### Abdominal pain

**Figure 4:**
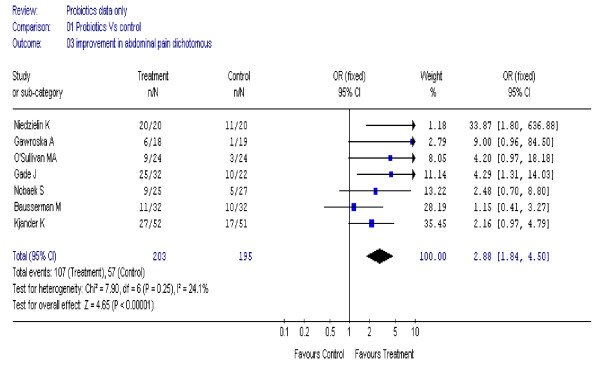
**Forest plot of improvement of abdominal pain (dichotomous data) in patients with IBS treated with probiotics compared to placebo**.

Seven trials [17-19,21,24,26,28] (398 participants) reported a statistically significant improvement in abdominal pain, OR 2.88 (95% CI, 1.84 to 4.50), I^2 ^= 24% (figure 4).

In five studies of adults only [18,21,24,26,28] (297 participants) the effect remained statistically significant, OR 3.34 (95% CI, 1.99 to 5.61), I^2 ^= 1%.

Two studies of children [17,19] (101 participants) reported on improvement in abdominal pain. However, high heterogeneity (I^2 ^= 63%) suggested pooling was inappropriate. In Bausserman's study [17] (64 participants), Lactobacillus GG (LGG) was not superior to placebo in relieving abdominal pain. There were no differences in other gastrointestinal symptoms, except for a lower incidence of perceived abdominal distension (P = 0.02 favouring LGG). In Gawroska's study [19] (37 participants), those in the LGG group were more likely to have treatment success (defined as no pain based on face pain scales) than those in the placebo group and had reduced frequency of pain (P = 0.02), but not severity of pain.

Using continuous data in nine trials [17,19-23,26,27,30] (792 participants) there was no statistically significant improvement in abdominal pain, SMD 0.05; (95% CI, -0.09 to 0.19), I^2 ^= 51%. The heterogeneity was high. The most likely reason for this was the use of different scales in different studies; some used Likert scales ranging from 0 to 6 whereas others used visual analogue scales or face pain scales.

### Flatulence

**Figure 5:**
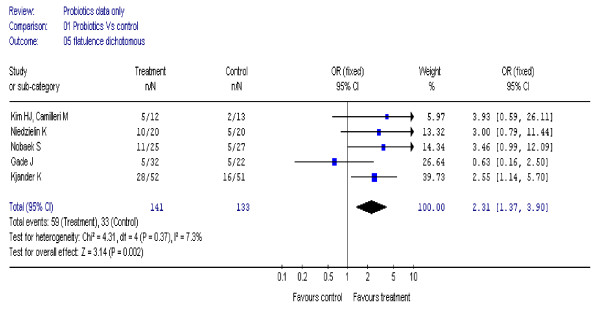
**Forest plot of improvement of flatulence (dichotomous data) in patients with IBS treated with probiotics compared to placebo**.

Five studies [18,21,22,24,26] (274 participants) reported a significant improvement in symptoms of flatulence, OR 2.31 (95%CI, 1.37 to 3.9), I^2 ^= 7% (figure 5). Using continuous data in five studies [21-23,26,30] (388 participants), there was no statistically significant improvement in symptoms of flatulence, SMD 0.11; (95% CI, -0.09 to 0.31), I^2 ^= 49%. The high heterogeneity was most likely to be due to use of different scales in different studies.

### Bloating

**Figure 6:**
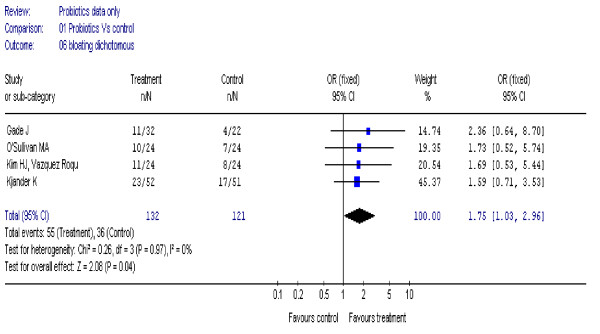
**Forest plot of improvement of bloating (dichotomous data) in patients with IBS treated with probiotics compared to placebo**.

Four studies [18,21,23,28] (253 participants) reported a statistically significant improvement in symptoms of bloating, OR 1.75 (95% CI, 1.03 to 2.96), I^2 ^= 0% (figure 6).

Using continuous data in six studies [20-23,27,30] (653 participants), there was no statistically significant difference in symptoms of bloating, SMD 0.05 (95% CI, – 0.10 to 0.21), I^2 ^= 8%.

### Quality of Life

Four trials aimed to report quality of life (QoL) but inadequate data prevented pooling of the results. In a study by O'Mahony et al [27] QoL was assessed by administration of an IBS specific questionnaire. They reported that for most domains, QoL scores were numerically lower than those for placebo for the patients randomized to the probiotics, but reached statistical significance versus placebo, during the treatment phase only, for health worry for bifidobacterium (at the 0.05 level) and dysphoria for lactobacillus at the 0.10 level. In Kajander's Study [21] the RAND 36 items health survey was used. They report that at baseline the mean QoL was somewhat higher in the probiotic group, but the difference between the groups was non significant compared with the baseline. There was no change in the mean score at three months or six months in either group.

Whorwell et al [30] used the IBS specific QoL questionnaire and the Hospital Anxiety and Depression Scale (HAD). No significant change in the QoL or HAD scores was reported with any of the probiotic dosages in comparison to placebo.

In a study by Guyonnet et al [20], health related QoL was assessed by the administration of the Functional Digestive Disorders Quality of Life questionnaire. They reported that the global score did not differ significantly between the probiotic group and the control group.

### Adverse effects

Nine trials reported that there were no adverse effects with probiotics [17-19,21-24,26,28], four trials reported adverse effects [20,25,27,30] and one study [29] reported no data.

In the Niv study [25] (54 participants), the following adverse events were reported in the probiotic group: dyspepsia (one), headache (one) and nausea (none) and in the control group: dyspepsia (three), headache (none) and nausea (one). The differences were not significant. In the Whorwell study [30] (362 participants), 17 of subjects withdrew because of an adverse event. There was no significant difference between the groups and there were no details on the characteristics of adverse events. In O'Mahony's study [27] (80 participants), four subjects reported adverse events during the study. In Guyonnet's study [20], (274 participants) 23 subjects (ten from the control group and 13 from the probiotic group) reported minor adverse events. Four in the control group and three in the probiotic group stopped the product after an adverse event. No further details were given on the nature of these events.

## Discussion

In patients with IBS, probiotics showed a modest improvement in overall symptoms, using both dichotomous and continuous data. However, it is interesting to note that neither of the two studies [20,30] which contributed most of the weight in the analysis, were statistically significant. It is likely that the two Bifidobacterium strains used in these two studies may have been ineffective. Based on average control event rates the Numbers Needed to Treat is estimated to be between 9 and 21 to have 1 patient improve. By removing studies with quality scores less than four, the results remained stable to sensitivity analysis.

For individual symptoms the results differed between the pooled dichotomous and pooled continuous data. Using dichotomous data probiotics also improved symptoms of abdominal pain, flatulence and bloating. However using continuous data, the improvement in these symptoms were not statistically significant. One study used a cross-over design [28]. The data presented allowed us to include it for dichotomous outcomes (bloating and abdominal pain). Excluding this study did not affect the findings for abdominal pain, but rendered the effect on bloating non-statistically significant. The heterogeneity was also high for symptoms of abdominal pain and flatulence. This is likely to be due to use of different scales in different studies.

Other therapies for IBS include antispasmodics, antidiarrhoeal agents, laxatives and antidepressants [1], but overall the evidence for efficacy of these existing drug therapies for IBS is weak [9]. Probiotics have been used previously in different conditions [10,11]. On entering the gastrointestinal tract, probiotics are unaffected by acid, bile salts and proteolytic enzymes. In the small bowel they multiply and live on the surface of epithelial cells. Their main beneficial effect is to act as a barrier to harmful organisms by adherence and production of substances that have an antibiotic effect, as well as stimulating immune processes in the host [11]. In the colon they have a major role of fermenting undigested carbohydrates and soluble dietary fibre, producing short-chain fatty acids [11]. Probiotics may change the flora, effecting the fermentation process, so less gases are produced that may cause symptoms, or they may interfere with the growth or harmful effect of producing diarrhoea, or they may stimulate the immune process to prevent some unidentified antigen response [34].

As there were inconsistencies in the reporting of adverse events, it was not possible to adequately estimate the frequency of the adverse events. However, nine studies reported that there were no adverse events. Clearer reporting of adverse events in future studies would be helpful. In addition, a review of probiotics in Crohn's Disease found they were generally well tolerated and few side effects were reported. The reported side effects included bloating, diarrhoea, constipation, nausea and epigastric pain [35]. Occasionally probiotics can cause infections in immunocompromised individuals [36,37].

One of the limitations of this review is exclusion of non-English language publications which may lead to an over-representation of positive studies. Other limitations include the clinical and statistical heterogeneity between studies. For example, the age and gender of the study populations varied as did the length of follow up and dosage and type of probiotics used. There was also variation in the definition of overall symptoms in different studies: two studies included only subcategories of the IBS population (constipation – [20] or diarrhea-predominant participants [22]. Studies varied in relation to the length of treatment, number, type, dosage and strengths of probiotic(s) used. Whilst some studies used one probiotic, others used a combination e.g. VSL#3 which contains a cocktail of 8 different bacterial strains. In three studies more than one treatment arm was used [27,29,30]. Whorwell et al [30] studied a probiotic (*B. infantis *35624) at three different strengths of 10^6^, 10^8^, 10^10 ^against placebo. The study found *B. infantis *35624 at a dosage level of 10^8 ^cfu is effective in reducing the symptoms of IBS at four weeks. They suggested that this may be because the highest dose formulation 1 × 10^10 ^cfu, "coagulated" into a firm glue like mass. As Whorwell's study had more than one intervention arm, in our meta-analysis a conservative estimate (i.e. the intervention with the least effect) was used. The majority of trials [11] lasted eight weeks or less. This is important in a condition that is chronic and usually intermittent. Therefore, longer term studies would be more helpful. In some trials, we could not include data as they were incomplete or missing despite writing to authors. Specifically, trials should report individual outcomes for IBS. These should include important symptoms such as urgency and difficult defecation. We could not carry out subgroup analysis based on sex and probiotics used. Problems due to different scales used occurred for reporting of continuous outcomes. For instance, some studies used Likert scales ranging from 0 to 6 whereas others used visual analogue scales and face pain scales. The use of different scales may explain high heterogeneity observed in pooling of continuous data for abdominal pain and flatulence. High heterogeneity, due to small number of participants, may also have occurred in the case of subgroup analysis of children. As the number of studies was small it was difficult to draw conclusions on publication bias. For overall assessment with the largest number of studies (seven studies), only one study with negative result was reported, raising the possibility that small studies with negative findings may not have reached publication. Finally, in this review flatulence was included despite not strictly being part of the IBS criteria. This was due to a number of early studies including flatulence as a symptom [18]. Our meta-analysis was carried out before the publication of a study by Kajander et al [38] published in 2008. This randomized placebo controlled trial using multispecies probiotic further strengthens our findings.

## Conclusion

Probiotics may have a role in alleviating some of the symptoms of IBS, a condition for which currently evidence of efficacy of drug therapies is weak. Longer term trials are recommended as IBS is a condition that is chronic and usually intermittent. However, further research should focus on the type, optimal dose of probiotics and the subgroups of patients who are likely to benefit the most.

## Competing interests

The authors declare that they have no competing interests.

## Authors' contributions

NH carried out the literature search, selection, validity assessment, data abstarction and data analysis. NH wrote the paper and incorporated the comments from other authors and peer reviewers. CH carried out the literature search, selection, validity assessment, data abstarction, cotributed to data analysis and writing of the paper and commented on all the drafts. KRM had the original idea for the paper, formulated the protocol and contributed to data abstraction and analysis. RP gave statistical advice, contributed to data abstraction and analysis and commented on various drafts. NR carried out the literature search and contributed to writing the section on the literature search. PG offered advice and commented on various drafts. All authors reviewed and approved the final draft of the paper.

## Pre-publication history

The pre-publication history for this paper can be accessed here:

http://www.biomedcentral.com/1471-230X/9/15/prepub
